# Increased expression of human endogenous retrovirus K in endomyocardial biopsies from patients with cardiomyopathy – a transcriptomics meta-analysis

**DOI:** 10.1186/s12864-024-10595-6

**Published:** 2024-07-20

**Authors:** Markus B. Heckmann, Daniel Finke, Leander Sauerbrey, Norbert Frey, Lorenz H. Lehmann

**Affiliations:** 1 Department for Cardiology, Angiology, and Pneumology, Heidelberg University Hospital, Heidelberg, Germany; 2 German Centre for Cardiovascular Research: DZHK, Partner Site Heidelberg/Mannheim, Heidelberg, Germany; 3 German Cancer Research Center (DKFZ), Heidelberg, Germany; 4 Center for Cardiovascular and Preventive Medicine, ATOS Klinik, Heidelberg, Germany

**Keywords:** Endogenous retrovirus K, Dilated cardiomyopathy, RNAseq, Viral expression

## Abstract

Most studied, investigating transcriptional changes in myocardial biopsies focus on human genes. However, the presence and potential consequence of persistent expression of viral genes within the myocardium is unclear. The aim of the study was to analyze viral gene expression in RNAseq data from endomyocardial biopsies. The NCBI Bioproject library was screened for published projects that included bulk RNA sequencing data from endomyocardial biopsies from both healthy and diseased patients with a sample size greater than 20. Diseased patients with hypertrophic, dilated, and ischemic cardiomyopathies were included. A total of 507 patients with 507 samples from 6 bioprojects were included and mapped to the human genome (hg38). Unmappable sequences were extracted and mapped to an artificial ‘super-virus’ genome comprising 12,182 curated viral reference genomes. Subsequently, the sequences were reiteratively permutated and mapped again to account for randomness. In total, sequences from 68 distinct viruses were found, all of which were potentially human pathogenic. No increase in cardiotropic viruses was found in patients with dilated cardiomyopathy. However, the expression levels of the particle forming human endogenous retrovirus K were significantly increased (q < 0.0003, ANOVA). Higher expression levels were associated with increased expression in mitochondrial pathways such as oxidative phosphorylation (*p* < 0.0001). In Conclusion, expression of human endogenous retrovirus K is significantly increased in patients with dilated cardiomyopathy, which in turn was associated with transcriptional alterations in major cellular pathways.

**Supplementary Information:**

The online version contains supplementary material available at 10.1186/s12864-024-10595-6.

## Introduction

With broad availability of sequencing data from myocardial biopsies, it is possible to assess transcriptional changes of coding genes in different cardiac pathologies, such as dilated or hypertrophic cardiomyopathy. Most studies investigate differentially expressed genes by comparing differences in human gene expression head-to-head. Assessing differentially expressed genes in the human myocardium relies on the measurement of ‘human genes’ as defined by the ‘Genome Reference Consortium’ while ‘non mappable reads’ are normally trashed. However, so far it seems to be unclear, if persistent viral gene expression is present in myocardial tissues (either by integration or by persistent viral transduction). Virus-associatedmyocarditis has been shown to directly impair cardiac function by targeting mitochondrial pathways [1]. Persistent enteroviral low-copy RNA expression has been attributed by some groups to unexplained dilated cardiomyopathy [2–5]. Bouin et al. found enteroviral RNA in autopsies of 33% of their 24-patient cohort [2]. Other studies with samples from explanted hearts from DCM patients showed a similar detection rate of enteroviral RNA or DNA from parvovirus B19 [6, 7]. However, autopsy studies from patients with unremarkable cardiac findings also show a high prevalence of parvovirus B19 [8]. The impact and frequency of persistent transcription from viral genes is less clear. In an unbiased and large-scale approach, we aimed to analyze viral gene expression in publicly available RNA-seq data from endomyocardial biopsies. We focused on non-human-mappable reads and mapped them to a’super-virus-genome' consisting of 12,182 publicly available virus genomes. By further statistical analysis we found an association between the expression of the endogenous retrovirus K and the presence of dilated cardiomyopathy.

## Materials and methods

### Search strategy and data acquisition

The *NCBI Bioproject* library was queried using the term *heart AND human by* applying the filters *transcriptome or gene expression* with available data and *mammals*. Projects with bulk RNA sequencing of left ventricular endomyocardial biopsies, which contained more than 20 persons comprising both diseased and healthy adult controls, were included.

### Raw data handling and mapping

All data analysis was performed using R (v. 4.3.0). RNAseq raw data were mapped to exons of the human genome Hg38 using Rsubread (v. 2.14.2) [9]. Only paired reads were counted. Counts were subsequently normalized with EdgeR (v. 3.42.4) [10], and reads per kilobase million (RPKM) were calculated to account for gene size and sequencing depth. Nonmappable sequences were extracted and used to map against reference sequences of 12,182 viral genomes. As a comparison, unmappable sequences were also mapped against 804 known human pathogenic viral reference genomes. An artificial genome comprising each reference sequence as an individual chromosome was created for this purpose using Rsubread. A complete list of accession numbers of the reference genomes, including clear names and sequence lengths, can be found in a supplemental csv file. To identify specific transcripts, we generated a Simplified Annotation Format (SAF) file from a General Feature Format (GFF3) file to label transcripts. Subsequently, we employed the 'featureCounts' method from the Rsubread package to identify paired reads that aligned with these annotated transcripts.

### Testing for randomness in viral mapping across and within each sample

To test against randomness, every unmappable string in the raw data was permutated in triplets by applying the Euler method using the Universalmotif (v. 1.18.1) and Biostrings (v. 2.68.1) packages [11, 12]. Important features such as sequencing depths, GC content, sequence variation, and number of sequences in each sequencing file were thus preserved. A permutated raw data file was stored for each sample and mapped to the viral reference genome. Counts were normalized to sequencing depth, and reference sequence length and RPKMs were calculated accordingly. With the information derived from counts from permutated and nonpermutated data, the MKPower (v 0.7) [13] package was used to simulate the sample size of permutations needed for each raw data file to test for significant differences using a Wilcoxon test with a significance level threshold of 1 × 10^–16^ to account for subsequent p level adjustment. Every additional permutation was mapped, and counts were generated.

For each virus found in the original dataset, the original sequencing counts were used as an estimate, and a single sample Wilcoxon signed-rank test was used to test for differences against the permutated data. The Holm‒Bonferroni method was used for p-level adjustment. Only mappings with increased counts in the original dataset and an adjusted p-level below 0.05 were considered nonrandom. Random counts were excluded from the analysis.

### Statistical analysis

Exploratory analyses were performed using plots and graphs created with ggubr [14]. Heatmaps were generated using *heatmap3* (v. 1.1.9). For each virus, an analysis of variance was performed to test for differences in expression between patients with dilated cardiomyopathy using the bioproject as a covariate. As many viruses were found in only a few samples, an analysis of deviance was also performed in the same manner. Proportions were compared with a Χ^2^-Test. False discovery rates (FDRs) were calculated using the Benjamini‒Hochberg algorithm. *P* values were adjusted using the Holm‒Bonferroni method. An adjusted *p* value or an FDR below 0.05 was considered significant.

### Gene set enrichment analysis

Correlation coefficients between viral and gene expression were calculated with Pearson’s method. Genes were ranked according to their correlation coefficient, and a gene set enrichment analysis was performed for KEGG and Reactome pathways using the *clusterfiler* (v. 4.8.2) and *ReactomePA* (v. 1.44.0) packages. [15, 16] Relevant KEGG pathways were drawn using the *pathview* package (v. 1.40.0). Enrichment results were plotted using the *enrichplot* package (v. 1.20.0).

## Results

In total, 359 projects were found in the *NCBI Bioproject* library. A total of 191 satisfied the applied filters, and 8 studies met the inclusion criteria: left ventricular endomyocardial biopsy, bulk sequencing data, and more than 20 individuals per project. One study (PRJNA633482) [17] with 29 individuals with LV biopsies, which was the only study using CAGE sequencing as opposed to bulk RNA sequencing, was excluded due to an imbalance in sample composition (20 healthy, 4 ICM, 3 DCM, 2 HFpEF). The remaining 7 projects were included in the analysis: PRJNA198165 [18], PRJNA209081 [19], PRJNA239241, PRJNA522931, PRJNA549848, PRJNA557232, and PRJNA595151. The search was last updated on July 27 2023.

### Study population and library mapping

A total of 530 individuals from 7 different projects were included in this analysis (see Table 1). The breakdown of characteristics by diagnosis and bioproject can be found in the supplements (Supplementary Table 1 and Supplementary Table 2). Fifty-nine percent of patients were male, aged 51 ± 14 years. (mean ± sd), predominantly male (59%). A total of 209 patients were diagnosed with dilated cardiomyopathy (DCM), 61 with hypertrophic cardiomyopathy (HCM) and with heart failure due to coronary artery disease (ischemic cardiomyopathy: ICM). The median library size was 10.3 gigabases [7.9–12.6, IQR]. A median of 3.0% [2.3–4.4, IQR] of the library size was not mapped to the human genome.

**Table 1:** Patient characteristics and dataset composition. DCM: dilated cardiomyopathy, HCM: hypertrophic cardiomyopathy, ICM: ischemic cardiomyopathy. SD: standard deviation. IQR: interquartile range

Total	*N* = 530
Age (mean (SD))	50.98 (13.89)
Sex = male (%)	260 (59.1)
Ethnicity (%)	
African American	122 (23.0)
Caucasian	233 (44.0)
Not reported	175 (33.0)
diagnosis (%)	
DCM	209 (39.4)
HCM	61 (11.5)
healthy	221 (41.7)
ICM	39 (7.4)
BioProject (%)	
PRJNA198165	24 (4.5)
PRJNA209081	30 (5.7)
PRJNA239241	36 (6.8)
PRJNA522931	35 (6.6)
PRJNA549848	23 (4.3)
PRJNA557232	27 (5.1)
PRJNA595151	355 (67.0)
library size [gigaBase] (median [IQR])	10.32 [7.87, 12.56]
unmapped [gigaBase] (median [IQR])	0.30 [0.18, 0.51]
Mapped/unmapping ratio [%] (median [IQR])	2.97 [2.25, 4.39]

### Mapping to viral genomes reveals nonrandom viral gene expression

**Fig. 1:**
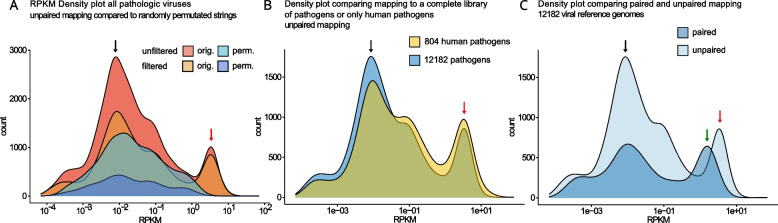
Mapping of viral data. An RPKM density plot comparing mapping results on original and permutated data for filtered and unfiltered data. Filtering reduced the counts in the permutated dataset as well as in the low RPKM peak (indicated by a black arrow) of the original dataset, while the high RPKM peak (indicated by a red arrow) was preserved. In our dataset, mapping to only human pathogenic or a wide variety of viral reference genomes did not tremendously change the overall expression of RPKMs (**B**). Paired mapping as opposed to unpaired mapping reduced the overall RPKMs and led to a minimal reduction in the high RPKM peak (red arrow) and a more pronounced reduction in the low RPKM peak (black arrow) (**C**) – this is the peak where most unpaired RPKMs from permutated files were located

To test whether nonmappable reads are sequencing artifacts or are based on virus expression, we generated random sequences (permutations) based on the nonmappable reads. These permutated reads were then tested for significant enrichment and compared to initial nonmapped sequences. Simulations showed that 133 to 136 permutations of the original sequencing strings were necessary to achieve the desired significance level. Viral gene expression was detected in 472/530 samples before filtering significant hits. After filtering, viral gene expression was found in 450/530 samples. RPKM was higher in the original files than in the permutated files even before filtering. Aside from reducing the number of positive samples, a relevant reduction in background noise was achieved (see Fig. 1A). Interestingly, the distribution of RPKMs did not change much depending on the reference genomes. Of note, all significant hits also represented human pathogens (Fig. 1B). Paired-end mapping reduced the number of low-expression RPKMs, notably the region where permutations reached high RPKMs, while conserving most high-expression RPKMs. As expected, RPKMs were lower for paired mapping than unpaired mapping. We compared virally mapped RNA from endomyocardial biopsies, analyzing transcript and genome counts. Both methods showed similar distribution patterns, with transcripts having slightly fewer counts (see supplementary Fig. 1). Only filtered paired mappings were used for further analyses.

### Batch effects and potential contamination

**Fig. 2:**
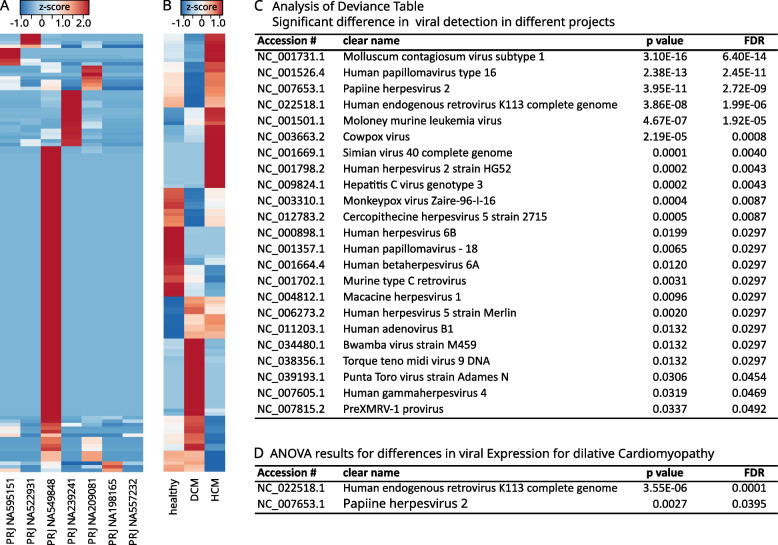
Batch effects and potential contamination.** A** and **B** show clustered heatmaps of viral gene expression according to the project (**A**) and diagnosis (**B**). Note the isolated expression of most of the viral genes in PRJNA549848 as an indicator of contamination. Analysis of deviance (ANDEVA, **C**) and analysis of variance (ANOVA, **D**) were performed after excluding this project. Viruses in **C** show differences in the frequency with which they were detected in different projects. Two viruses showed higher expression levels in dilated cardiomyopathy (**D**)

In our initial analysis and visualization through clustered heatmaps, we observed that among the 206 viruses identified, 138 (67%) were exclusively linked to a single project, PRJNA549848. On average, 34 distinct viruses were found in each sample in this project, compared to an average of 1.8 (1.4–2.1 IQR) in the remaining projects. Despite the limited number of projects analyzed, this discrepancy was statistically significant (*p* = 0.0313, Wilcoxon test). Figure 2A and B show heatmaps with viral gene expression according to bioproject and disease state. Project PRJNA549848 (*n* = 23) was excluded from further analyses as an outlier Project. The reasons for the observed differences might be contamination during sample preparation or differences in sample handling and storage conditions.

To further investigate the correlation of the different projects and dilated cardiomyopathy with viral gene expression, an analysis of deviance (ANDEVA) was performed using both as covariates. After *p* value adjustment for multiple testing, 23 of the remaining 68 viruses were significantly more abundant in one or the other project. The top hit, ‘Molluscum contagiosum’ (*p* = 3.1 × 10E-16), might well be explained by contamination of the samples during handling, since this virus usually resides in human epidermal cells. Contamination from other nonhuman samples might have caused the Moloney murine leukemia virus to spike in a few samples. Contamination from other sequencing experiments recently sequenced in the same facility, or even—and most interestingly—a different regional background, might explain differences detected in the almost ubiquitously detected human endogenous retrovirus K. The exact reasons remain speculative. However, the expression level of the virus found might provide some insight, as contamination tends to be small. A further insight gives the ANOVA performed in Fig. 1D showing a significantly increased expression of the human endogenous retrovirus K as well as the Papiine herpesvirus 2 in myocardial biopsies from patients with dilated cardiomyopathy.

**Fig. 3:**
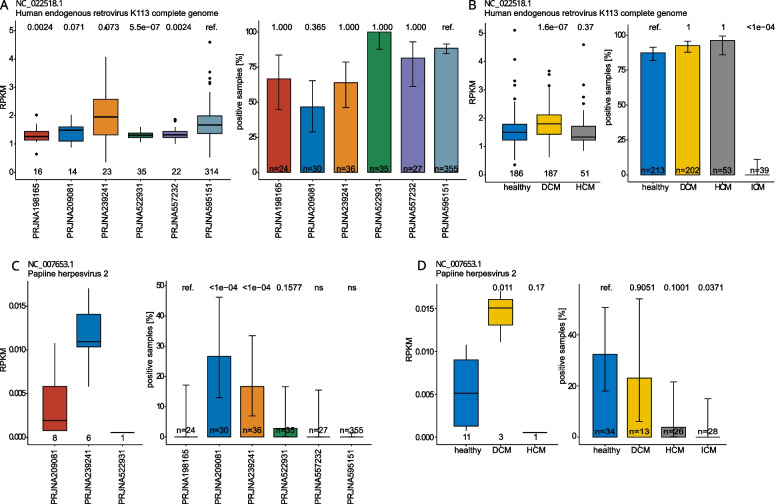
Viral detection of papillomavirus 2 and the human endogenous retrovirus K across the different bioprojects and in different patient populations. The human endogenous retrovirus shows a robust expression level across all projects and patient populations (**A**, **B**). A small but significant increase in viral expression was detected in patients with dilated cardiomyopathy (DCM). Papiine herpesvirus was detected at an overall low level in only 15 patients in 3 projects, of which only 3 were diseased, possibly explained by contamination (**C**, **D**)

We further analyzed the distribution of the virus across the different projects and diagnoses. Papiine herpesvirus 2 was detected only in 15 samples from 3 projects. Given the low expression of the virus (compare Fig. 3A to C) and the inconsistent detection across different projects (see Fig. 3 C and D), contamination seems more probable. The human endogenous retrovirus K113 shows a consistently high expression level across all bioprojects, with a further increase in patients suffering from dilated cardiomyopathy (see Fig. 3 A and B). Raw counts and RPKMs showed similar results for HERV K expression in different patient populations—see supplementary Fig. 2.

All HERV K113 RNA transcripts mapped to the Q779_gp1 gene, a protein coding gene of the putative env protein of HER K113. A blast analysis revealed this gene's extensive conservation across HERV-K subviruses, suggesting potentially the combined detection of env transcripts from other HERV-K-subtypes – see supplementary data file Table 4.

### No significant difference in the detection of cardiotropic viruses

We also analysed the detection rate of cardiotropic viruses (parvovirus B19, cytomegalovirus, human herpes virus type 6 as well as adeno- and enteroviridae) and did not find significant differences in detection rates between patients suffering from dilated cardiomyopathy or healthy individuals. No significant difference in cardiotropic viral RPKMs was detected between healthy and diseased individuals (see supplementary File).

### Expression levels of endogenous retroviral RNA correlate with specific upregulation and downregulation of pathways

**Fig. 4:**
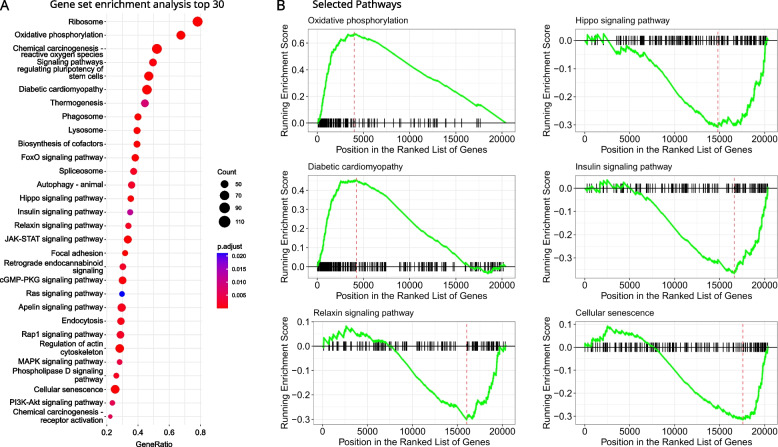
Gene set enrichment analysis derived from correlating viral expression levels of human endogenous retrovirus K with gene expression. In all patients, HER-K expression was correlated with major mitochondrial metabolic pathways and essential cellular signaling pathways **A**. GSEA plots of selected pathways are depicted in **B**. Correlation results for the pathways were also mapped to canonical KEGG images and can be downloaded from a supplemental zipped archive

To understand the changes associated with the increase in viral expression levels of human endogenous retrovirus K (HERV-K) in patients with dilated cardiomyopathy, we conducted a gene set enrichment analysis. This analysis showed a significant correlation of viral expression levels of HERV-K with genes involved in oxidative phosphorylation, Hippo signaling, insulin signaling, and other key metabolic and signaling pathways (see Fig. 4).

Higher expression levels of HERV-K were found in patients with DCM in our dataset (see Fig. 3B). HERV-K expression was positively correlated with genes involved in oxidative phosphorylation and diabetic cardiomyopathy and negatively correlated with genes involved in Hippo and insulin signaling, cellular senescence, and relaxin signaling (Fig. 4).

Interestingly, HERV-K expression was inversely correlated with cellular viral response mechanisms. A reactome pathway enrichment found an increased association with viral mRNA translation (R-HSA-192823, normalized enrichment Score (NES) 3.73, FDR < 0.0001) and viral RNA transcription and replication (R-HSA-168273, NES 3.33, FDR < 0.0001), whereas a negative association with IFN-stimulated genes (R-HSA-1169410, NES -0.33, FDR 0.0413) and ISG15 antiviral mechanism (R-HSA-1169408, NES -0.34, FDR 0.0326) was detected. The complete pathway analyses can be found in the supplementary file.

## Discussion

This is the first study to systematically analyze available RNAseq data of endomyocardial biopsies for the presence of viral RNA expression. In this work, we demonstrate that viral RNA can be successfully mapped in human endomyocardial biopsies. We reduced background noise by permutating strings and mapping paired reads. Our mapping results were not random but in many cases most likely explained by contamination.

Another meta-analysis on whole genome data found significant contamination and possible mapping errors mapping with the microbiome [20]. Certain mapped bacterial strains exhibited a strong correlation with human sex, which was attributable to incomplete reference sequences of the human Y chromosome and subsequent mismapping to bacterial strains. This study did not report mis-mapping of viral sequences. Contamination with human sequences seems to be a problem in bacterial reference sequences, leading to scrutiny of previously published studies on the cancer microbiome [21].

The main contributors to the viral DNA found in the abovementioned study were lambda phage and Epstein‒Barr virus [20]. Human herpesviruses 6A, 6B, and 7 exhibited great intersample variance, which was attributed to possible latent or active infection and possibly inherited chromosomally integrated human herpesvirus [20]. The main contributor to viral gene expression in our dataset was HER-K. In this regard, RNA expression and genomic sequencing differ greatly.

In contrast to previous reports, we found no evidence of a significant enrichment for enteroviral RNA expression in patients with DCM in the data [2, 3]. While murine animal models of protracted enteroviral expression exist, the clinical relevance of persistent very low copy enteroviral RNA and protein expression in human hearts months to years after infection is still unclear. Data from previous studies led us to hypothesize that Coxsackie B or parvovirus B19 are found in a subset of DCM patients [6, 7]. In our meta-analysis, we did not find an increased detection rate or expression levels of cardiotropic viruses in diseased patients. This, however, does not rule out that a transient viral infection, which was not detectable at the timepoint of the withdrawal of the endomyocardial biopsy, led to myocarditis that might have caused the DCM in some patients. The high detection rate of cardiotropic viruses in patients with unremarkable cardiac autopsy findings together with our analysis should trigger revisiting the relevance of chronic low copy cardiac viral expression [8].

As an alternative mechanism, the release of cardiac epitopes (e.g., troponine I) during an acute viral myocardial infection could trigger a long-lasting autoimmune response, as suspected in immune checkpoint inhibitor-induced myocarditis [22, 23]. The case of fulminant acute myocarditis during a parvovirus B19 infection without the myocardial detection of viral epitopes anecdotally underlines that viral myocardial expression is not mandatory for virally induced myocarditis [24]. Furthermore, the positive results for immunosuppressive therapy in acute viral myocarditis suggest the predominant autoimmune component of this disease [25, 26].

The detection of cardiotropic viruses in both healthy and diseased myocardium implies that additional factors are likely involved in the progression from viral presence to clinical disease. For instance, while low copy numbers of viral RNA might be detected in endomyocardial biopsies, this does not necessarily indicate an active pathogenic process. Our results highlight the importance of considering other potential etiologies, such as autoimmune mechanisms or genetic susceptibilities, when diagnosing and treating patients with DCM.

Furthermore, these findings underscore the necessity for more extensive research to validate the observed patterns. Mechanistic studies exploring the interactions between viral persistence, immune response, and genetic factors will be crucial in elucidating the complex pathogenesis of DCM. Such research could lead to improved diagnostic criteria and more targeted therapeutic strategies, ultimately enhancing patient outcomes.

Aside from this negative finding, we found the human endogenous retrovirus K to be increased in DCM. HERV-K is part of the HERV-K family. A family of endogenous retroviruses that first entered the human germ line several million years ago. HERV-K has an estimated entrance age of 200,000 to 450,000 years and is the youngest member [27]. In an initial British sample, 9 out of 31 patients tested positive, which led to an estimated prevalence of approximately 30% [27]. In the included projects, the positivity rate was significantly different among the samples (q = 1.9 × 10^–6^, ANDEVA) and ranged between 47% [29%;65%, 95% CI] and 100% [88%;100%, 95% CI]. HERV-K shows low expression in human samples and has the capacity to produce viral particles [28]. It has been proposed to be associated with autoimmune disease [29], but this link has not been consistently established [30]. In our meta-dataset, HERV-K RNA expression was associated with gene expression in key metabolic and signaling pathways. Although HERV-K RNA expression was increased in DCM patients, we found a positive correlation with mitochondrial pathways, which are negatively correlated with heart failure in preclinical studies [31]. However, it remains unclear whether upregulation of these pathways is a biological consequence of HERV-K expression or a maladaptive response. Further research is needed to validate these findings in other sequencing studies and in preclinical studies to reveal the potential mechanistic interactions of HERV-K with mitochondria.

The method described above – with its limitations – can be used to establish viral gene expression patterns for different human diseases. Analyzing the *viriome* in different human diseases might open a route to predict the clinical course of patients with DCM or an individual altered immune activity against the heart.

### Supplementary Information

Supplementary Material 1.

Supplementary Material 2.

Supplementary Material 3.

## Data Availability

The count-data and calculated RPKMs can be downloaded as a csv-File in the supplements with a reference to the already published Sequence Read Archive (SRA) Run Accession number (SRR) as well as the NCBI accession number of the virus. The R scripts will be shared upon request. The original data from the included projects can be downloaded from https://ncbi.nlm.nih.gov/bioproject/198165, https://ncbi.nlm.nih.gov/bioproject/209081, https://ncbi.nlm.nih.gov/bioproject/239241, https://ncbi.nlm.nih.gov/bioproject/522931, https://ncbi.nlm.nih.gov/bioproject/549848, https://ncbi.nlm.nih.gov/bioproject/557232, https://ncbi.nlm.nih.gov/bioproject/595151
