# Supplementary material for: Increased expression of human endogenous retrovirus K in endomyocardial biopsies from patients with cardiomyopathy – a transcriptomics meta-analysis
Source: BMC Genomics. 2024 Jul 20;25:707. doi: 10.1186/s12864-024-10595-6 (PMC11264874; doi:10.1186/s12864-024-10595-6)
Supplement: Supplementary file 2 — Supplementary Material 2. [file 12864_2024_10595_MOESM2_ESM.docx]

# Supplementary Tables

## Supplementary Table 1

**Supplementary Table 1.** Patient characteristics and dataset composition by Bioproject. DCM: dilative cardiomyopathy, HCM: hypertrophic cardiomyopathy, ICM: ischemic cardiomyopathy. SD: standard deviation. IQR: interquartile range.

|  | PRJNA198165 | PRJNA209081 | PRJNA239241 | PRJNA522931 | PRJNA549848 | PRJNA557232 | PRJNA595151 | p |
| --- | --- | --- | --- | --- | --- | --- | --- | --- |
| n | 24 | 30 | 36 | 35 | 23 | 27 | 355 |  |
| Age (mean (SD)) | Not reported | Not reported | Not reported | 34.17 (7.82) | Not reported | 37.56 (13.03) | 53.66 (12.62) | <0.001 |
| sex = male (%) | 0 | 0 | 0 | 26 ( 74.3) | 23 (100.0) | 21 ( 77.8) | 190 (53.5) |  |
| Ethnicity (%) |  |  |  |  |  |  |  | <0.001 |
| African American | 0 ( 0.0) | 0 ( 0.0) | 0 ( 0.0) | 0 ( 0.0) | 0 ( 0.0) | 0 ( 0.0) | 122 (34.4) |  |
| Caucasian | 0 ( 0.0) | 0 ( 0.0) | 0 ( 0.0) | 0 ( 0.0) | 0 ( 0.0) | 0 ( 0.0) | 233 (65.6) |  |
| not reported | 24 (100.0) | 30 (100.0) | 36 (100.0) | 35 (100.0) | 23 (100.0) | 27 (100.0) | 0 ( 0.0) |  |
| diagnosis (%) |  |  |  |  |  |  |  | <0.001 |
| DCM | 8 ( 33.3) | 0 ( 0.0) | 13 ( 36.1) | 0 ( 0.0) | 7 ( 30.4) | 15 ( 55.6) | 166 (46.8) |  |
| HCM | 0 ( 0.0) | 0 ( 0.0) | 0 ( 0.0) | 26 ( 74.3) | 8 ( 34.8) | 0 ( 0.0) | 27 ( 7.6) |  |
| healthy | 8 ( 33.3) | 15 ( 50.0) | 10 ( 27.8) | 9 ( 25.7) | 8 ( 34.8) | 9 ( 33.3) | 162 (45.6) |  |
| ICM | 8 ( 33.3) | 15 ( 50.0) | 13 ( 36.1) | 0 ( 0.0) | 0 ( 0.0) | 3 ( 11.1) | 0 ( 0.0) |  |
| library size [gigaBase] (median [IQR]) | 2.63 [2.12, 3.69] | 1.27 [1.14, 1.32] | 0.30 [0.26, 0.36] | 17.40 [15.68, 18.69] | 1.67 [1.25, 1.74] | 10.89 [10.52, 11.81] | 11.06 [9.42, 12.95] | <0.001 |
| unmapped bases  [gigaBase]  (median [IQR]) | 0.05 [0.04, 0.06] | 0.01 [0.01, 0.01] | 0.09 [0.06, 0.12] | 0.57 [0.50, 0.61] | 0.04 [0.04, 0.05] | 0.26 [0.23, 0.28] | 0.36 [0.26, 0.57] | <0.001 |
| mapping ratio [%] (median [IQR]) | 1.70 [1.61, 1.84] | 1.07 [0.94, 1.22] | 29.75 [26.09, 33.03] | 3.16 [2.96, 3.46] | 2.72 [2.54, 3.15] | 2.25 [2.14, 2.47] | 3.15 [2.44, 4.54] | <0.001 |

## Supplementary Table 2

**Supplementary Table 3.** Patient characteristics and dataset composition by Disease. DCM: dilative cardiomyopathy, HCM: hypertrophic cardiomyopathy, ICM: ischemic cardiomyopathy. SD: standard deviation. IQR: interquartile range.

|  | DCM | HCM | healthy | ICM | p |
| --- | --- | --- | --- | --- | --- |
| n | 209 | 61 | 221 | 39 |  |
| Age (mean (SD)) | 50.58 (12.18) | 41.11 (13.32) | 54.42 (14.31) | 42.33 (7.23) | <0.001 |
| sex = male (%) | 116 (61.7) | 42 (68.9) | 99 (52.7) | 3 (100.0) | 0.042 |
| Ethnicity (%) |  |  |  |  | <0.001 |
| African American | 77 (36.8) | 1 ( 1.6) | 44 (19.9) | 0 ( 0.0) |  |
| Caucasian | 89 (42.6) | 26 (42.6) | 118 (53.4) | 0 ( 0.0) |  |
| not reported | 43 (20.6) | 34 (55.7) | 59 (26.7) | 39 (100.0) |  |
| BioProject (%) |  |  |  |  | <0.001 |
| PRJNA198165 | 8 ( 3.8) | 0 ( 0.0) | 8 ( 3.6) | 8 ( 20.5) |  |
| PRJNA209081 | 0 ( 0.0) | 0 ( 0.0) | 15 ( 6.8) | 15 ( 38.5) |  |
| PRJNA239241 | 13 ( 6.2) | 0 ( 0.0) | 10 ( 4.5) | 13 ( 33.3) |  |
| PRJNA522931 | 0 ( 0.0) | 26 (42.6) | 9 ( 4.1) | 0 ( 0.0) |  |
| PRJNA549848 | 7 ( 3.3) | 8 (13.1) | 8 ( 3.6) | 0 ( 0.0) |  |
| PRJNA557232 | 15 ( 7.2) | 0 ( 0.0) | 9 ( 4.1) | 3 ( 7.7) |  |
| PRJNA595151 | 166 (79.4) | 27 (44.3) | 162 (73.3) | 0 ( 0.0) |  |
| library size [gigaBase]  (median [IQR]) | 10.28 [8.70, 12.19] | 13.25 [9.47, 16.38] | 10.77 [8.14, 12.58] | 1.23 [0.39, 1.84] | <0.001 |
| unmapped bases[gigaBase]  (median [IQR]) | 0.32 [0.22, 0.55] | 0.48 [0.25, 0.58] | 0.29 [0.18, 0.48] | 0.06 [0.01, 0.11] | <0.001 |
| mapping ratio [%] (median [IQR]) | 3.29 [2.43, 4.92] | 3.27 [2.95, 3.86] | 2.68 [2.17, 3.92] | 1.80 [1.27, 26.92] | <0.001 |

# Supplementary Figures

## Supplementary Figure 1

**
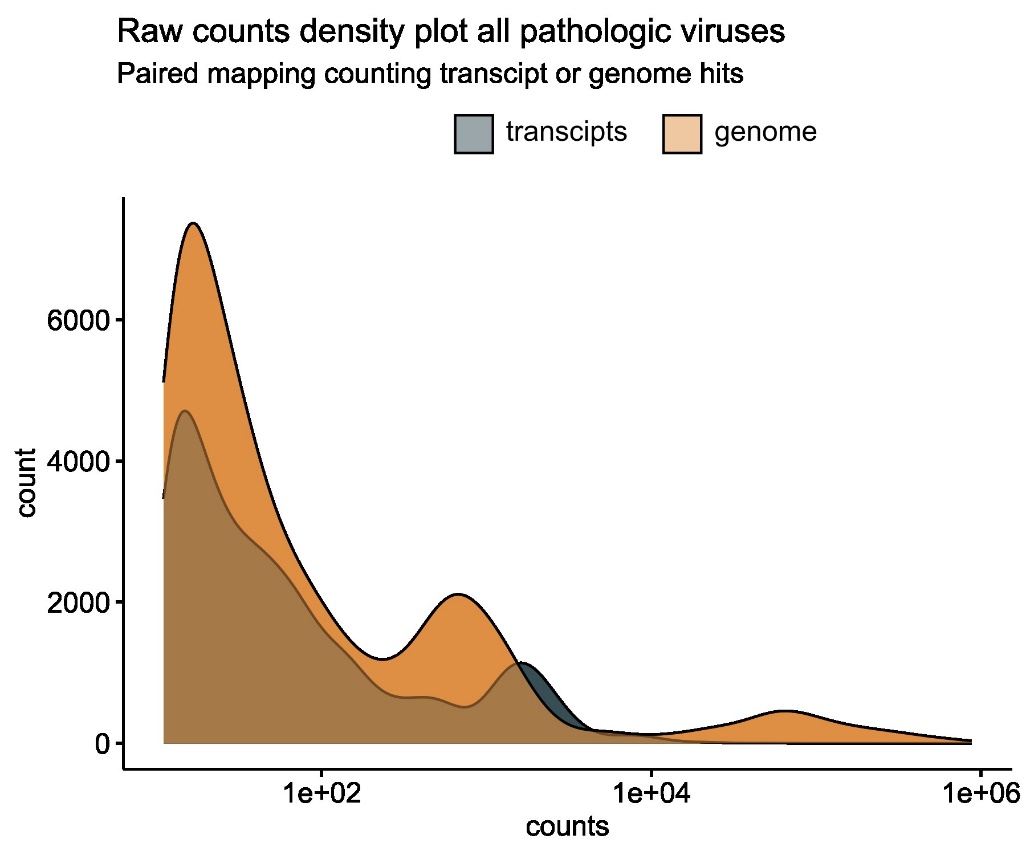
**

**Supplementary Figure 1.** Comparison of density plots for transcript counts and genome counts of virally mapped RNA from endomyocardial biopsies. Please note the same distribution pattern for both methods with a slightly less overall counts for transcripts.

## Supplementary Figure 2

**
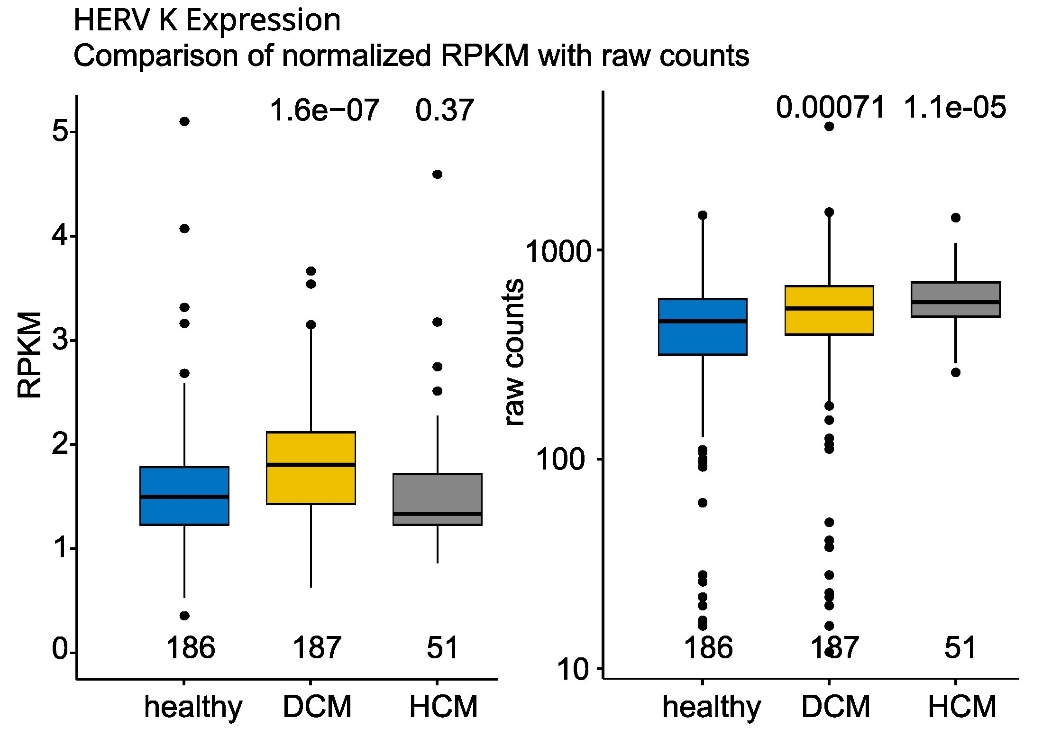
**

**Supplementary Figure 2.** Comparison of normalized RPKM and raw counts for HERV mapped transcripts.

## Supplementary Files

**Supplementary File 1.** **Name: KEGGPathwaysImagesFigure4.zip.** Zip archive containing the KEGG pathways pertaining to the enrichment plots of Figure 4 depicting the correlation coefficient of each gene with HER-K expression. The images are stored as PNG-Files.

**Supplementary File 2. Name: SupplementaryResults.xlsx.** This Excel File contains: **1.** A run table reporting patient and sample characteristics retrieved from the NCBI Bioproject library **2.** A table reporting the accession number, clear name and genome length of each virus included in the viral super genome. **3.** Every non-randomly mapped virus with its RPKM, raw counts, and details about the sample in which it was mapped as well as the p value and FDR of the Wilcoxon test performed on the permutations. **4.** BLAST results aligning the HERV K113 env gene reference sequence (NCBI Gene ID: 5141055, Gene Name: Q779_gp1) to known viral sequences. **5.** Viral detection and expression of cardiotropic viruses. **6**. Reactome enrichment analysis. **7**. KEGG Pathway enrichment analysis.
