# Supplementary figures and images for: Increased expression of human endogenous retrovirus K in endomyocardial biopsies from patients with cardiomyopathy – a transcriptomics meta-analysis
Source: BMC Genomics. 2024 Jul 20;25:707. doi: 10.1186/s12864-024-10595-6 (PMC11264874; doi:10.1186/s12864-024-10595-6)

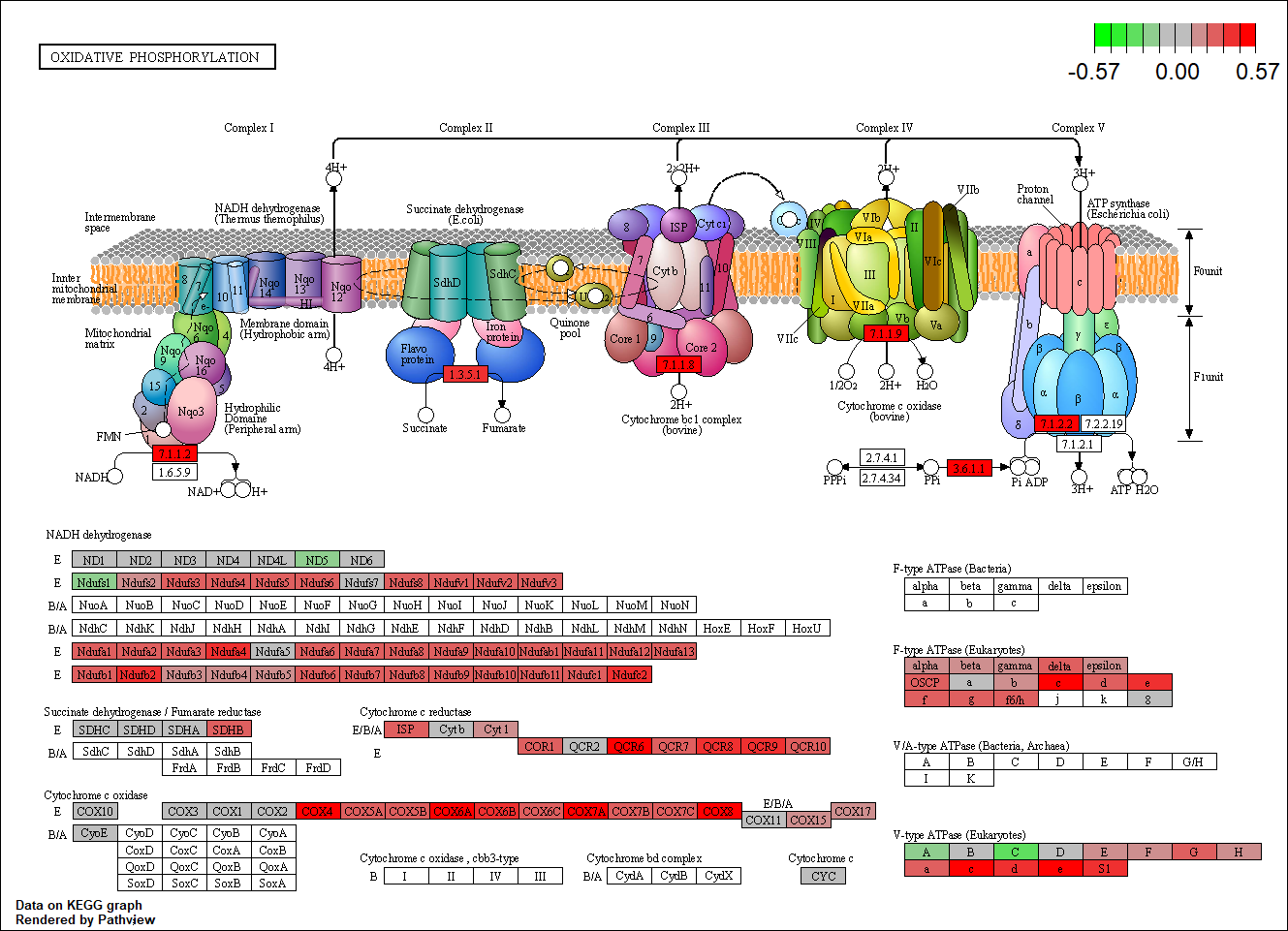

Supplement: Supplementary file 3 — Supplementary Material 3. [file 12864_2024_10595_MOESM3_ESM.zip › Figure4_KEGG_PathviewFiles/hsa00190.pathview.png]

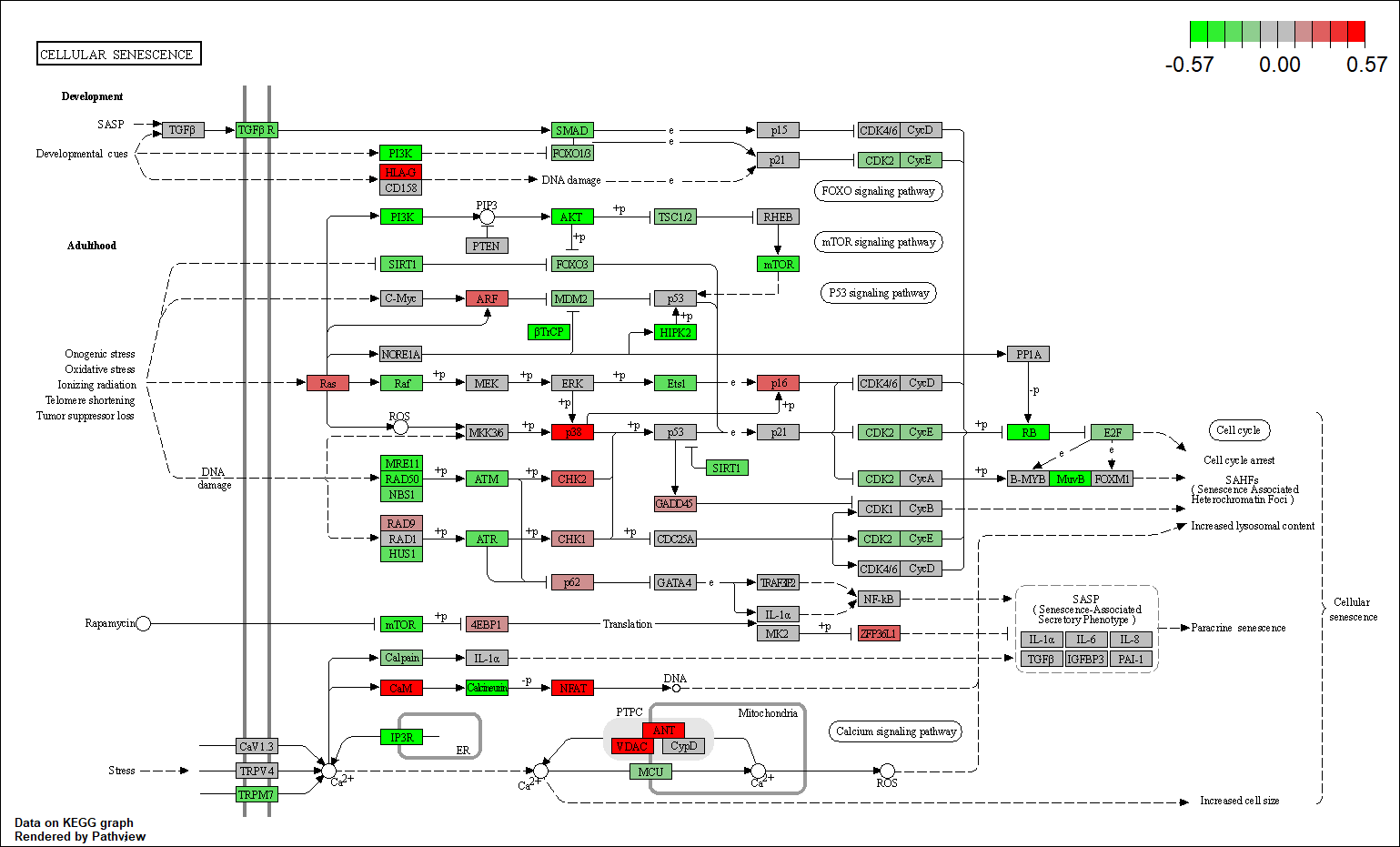

Supplement: Supplementary file 3 — Supplementary Material 3. [file 12864_2024_10595_MOESM3_ESM.zip › Figure4_KEGG_PathviewFiles/hsa04218.pathview.png]

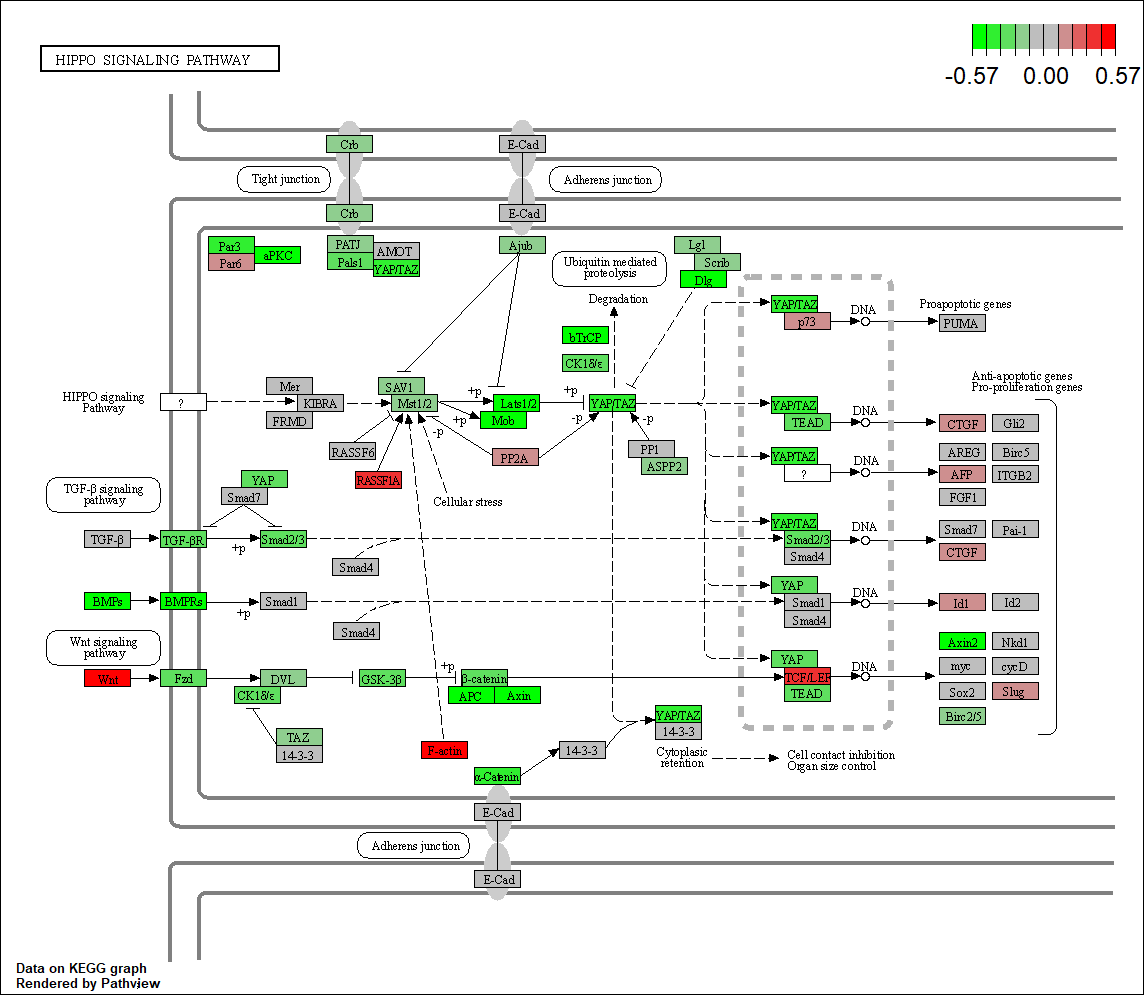

Supplement: Supplementary file 3 — Supplementary Material 3. [file 12864_2024_10595_MOESM3_ESM.zip › Figure4_KEGG_PathviewFiles/hsa04390.pathview.png]

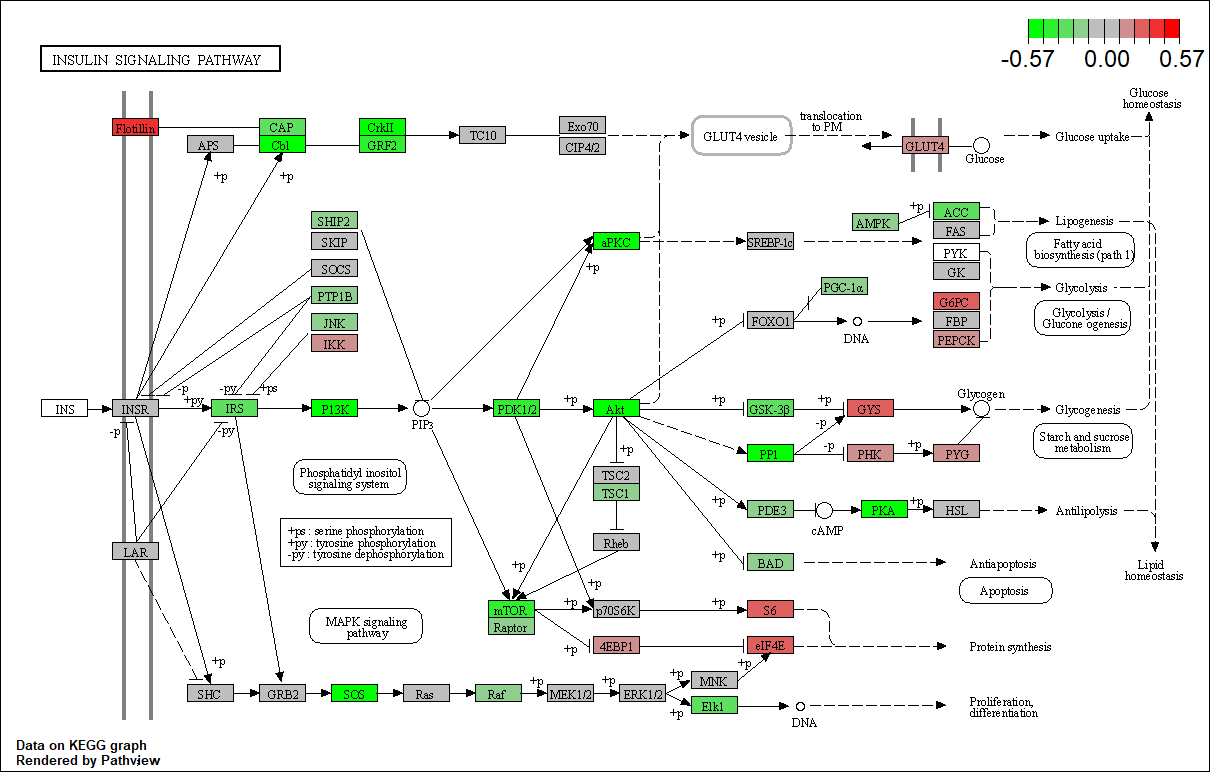

Supplement: Supplementary file 3 — Supplementary Material 3. [file 12864_2024_10595_MOESM3_ESM.zip › Figure4_KEGG_PathviewFiles/hsa04910.pathview.png]

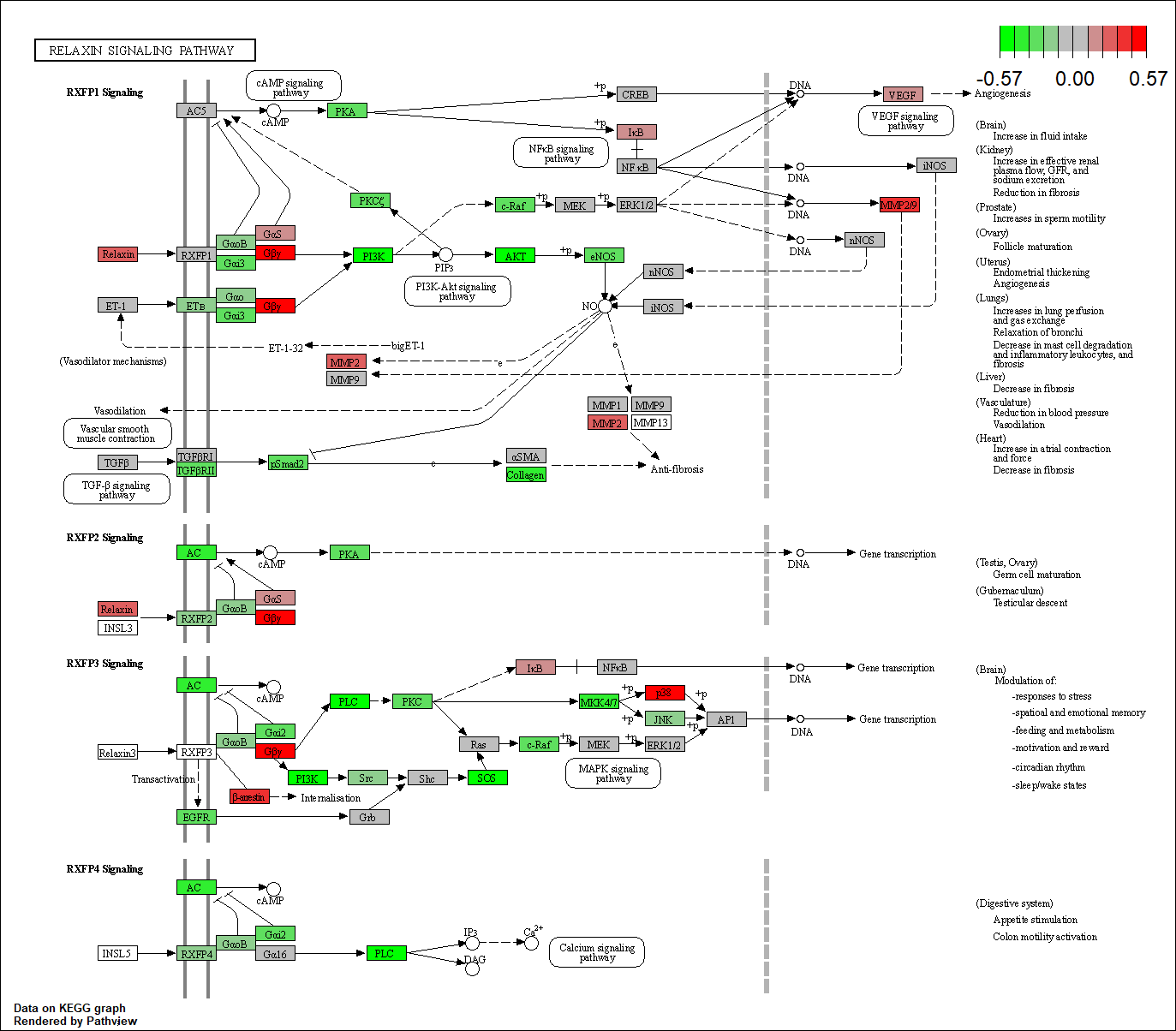

Supplement: Supplementary file 3 — Supplementary Material 3. [file 12864_2024_10595_MOESM3_ESM.zip › Figure4_KEGG_PathviewFiles/hsa04926.pathview.png]

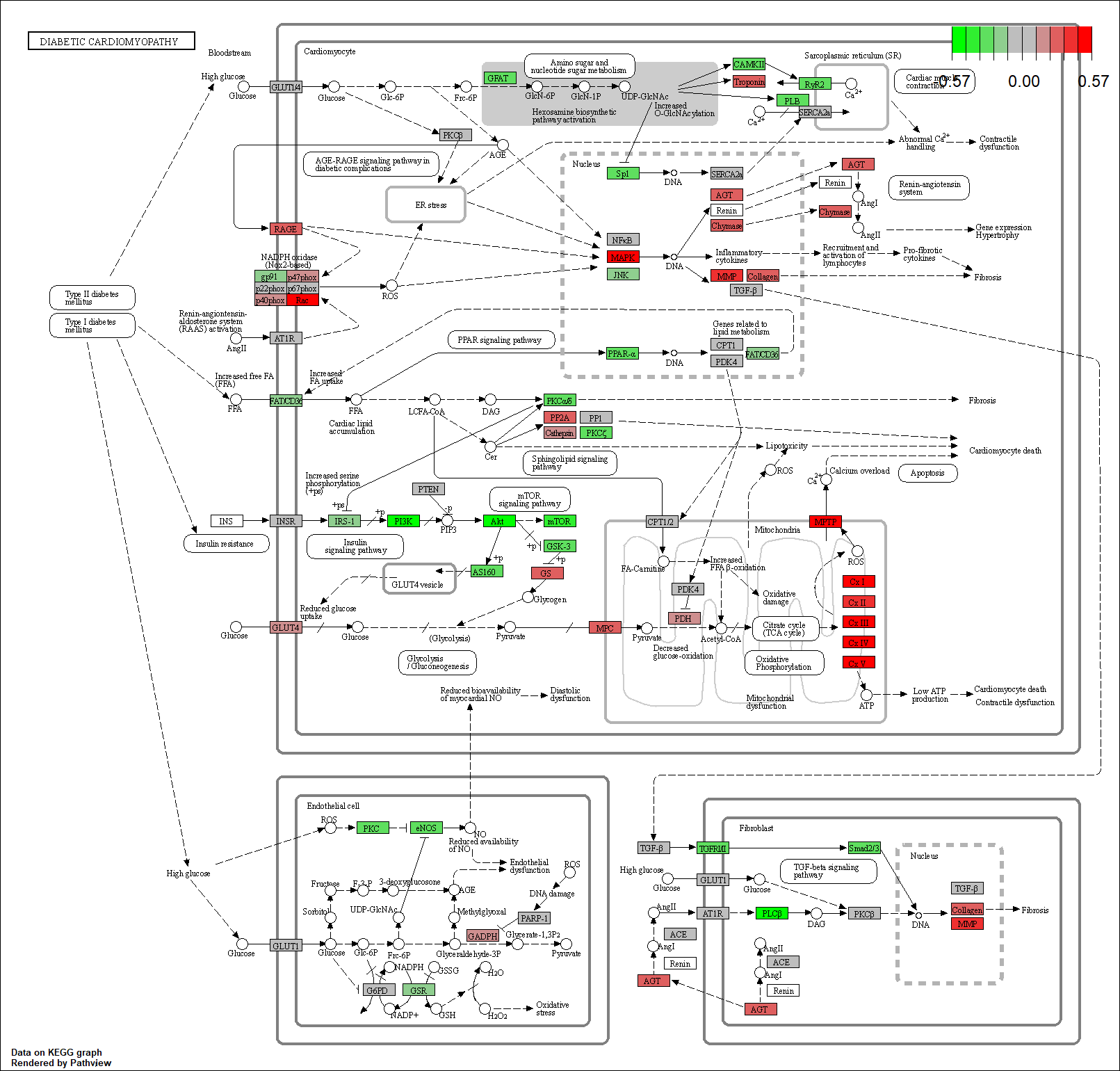

Supplement: Supplementary file 3 — Supplementary Material 3. [file 12864_2024_10595_MOESM3_ESM.zip › Figure4_KEGG_PathviewFiles/hsa05415.pathview.png]
